# Identification of Multiple Metabolic Enzymes from Mice Cochleae Tissue Using a Novel Functional Proteomics Technology

**DOI:** 10.1371/journal.pone.0121826

**Published:** 2015-03-26

**Authors:** David L. Wang, Hui Li, Ruqiang Liang, Jianxin Bao

**Affiliations:** 1 Department of Biology, Vanderbilt University, Nashville, TN, United States of America; 2 Department of Otolaryngology, School of Medicine, Washington University, St. Louis, MO, United States of America; Università della Calabria, ITALY

## Abstract

A new type of technology in proteomics was developed in order to separate a complex protein mixture and analyze protein functions systematically. The technology combines the ability of two-dimensional gel electrophoresis (2-DE) to separate proteins with a protein elution plate (PEP) to recover active proteins for functional analysis and mass spectrometry (MS)-based identification. In order to demonstrate the feasibility of this functional proteomics approach, NADH and NADPH-dependent oxidases, major redox enzyme families, were identified from mice cochlear tissue after a specific drug treatment. By comparing the enzymatic activity between mice that were treated with a drug and a control group significant changes were observed. Using MS, five NADH-dependent oxidases were identified that showed highly altered enzymatic activities due to the drug treatment. In essence, the PEP technology allows for a systematic analysis of a large enzyme family from a complex proteome, providing insights in understanding the mechanism of drug treatment.

## Introduction

Proteins play essential roles in numerous biological processes. Defining their roles and understanding their interaction networks are important in the discovery of basic biological processes and elucidation of important disease mechanisms. Over 20% of human genes code for enzymes which are responsible for most cellular biochemical processes [1]. Despite their importance, only a limited portion of the enzymes in the human genome have been characterized. Traditional studies of enzymes were carried out on either individual proteins or a small number of proteins which limits the ability to systematically understand the roles of these proteins in various biological functions.

Recently, new analytical technologies in genomics and proteomics have made it possible to systematically study gene regulation, protein expression and post-translational modifications on large scale. In genomics, gene expressions can be analyzed with a gene chip or other high throughput sequencing technologies. However, for certain genes, there is a weak correlation between their expression levels and their respective protein expression levels [2–5]. In proteomics, recent advancement in mass spectrometry allows one to systematically study protein posttranslational modifications and determine protein abundance [6–9]. However, this type of analysis of protein functions from a complex proteome is still a work in progress. For system biology and drug discovery studies, it is useful if a family of proteins can be isolated, identified, and their functions analyzed from the same samples. One area that systematic analysis of protein functions will bring value to is oxidative signaling pathways as they are involved in many normal and abnormal biological processes and have become focused areas for anti-cancer drug development [10].

Recent studies have reported various approaches to study protein functions systematically. For instance, a mechanism-based approach was used to profile various enzyme families [11,12]. In this approach, chemically synthesized probes were used to study enzyme families such as serine hydrolases, cysteine proteases, and tyrosine phosphatases and in conjunction with mass spectrometry and microarrays, this method provided interesting results in the study of various diseases. In another study, various chemical approaches such as combinatorial chemistry, fragment-based assembly and click chemistry were used with a microarray platform to study protein kinases, phosphatases and proteases [13]. All these approaches included the use of a complex protein mixture where proteins with high affinity toward the probes were isolated and further identified with mass spectrometry or individually expressed proteins were used to build a protein microarray which was later incubated with small molecule probes to study the corresponding enzymes. However, one challenge for the probe based approach is the design of specific molecules that have sufficient affinity toward the intended enzymes since the further characterization of the corresponding enzymes depend on the selective binding of the enzymes with the probes [14].

In our approach, proteins from a complex proteome were first separated using a 2-DE followed by electroelution to a specially designed Protein Elution Plate (PEP) in which proteins were separated into individual fractions based on their isoelectric points and molecular weights. After electroelution, proteins in each specific PEP well were transferred to a master microplate where a fraction of the sample was used for an enzyme assay. For specific fractions with an enzymatic activity of interest, mass spectrometry was used for further protein identification. This current study focused on oxidative enzymes, especially NADH and NADPH-dependent oxidases because in the cochlea, several NADH and NADPH-dependent oxidases have been implicated in hearing disorders such as age-related and noise-induced hearing loss [15]. By using this new method, we have identified a subset of oxidases in mouse cochleae that exhibit dramatically altered activities after a drug treatment against noise-induced hearing loss [16]. The data provided proof-of-concept for this novel functional proteomics technology.

## Materials and Methods

### Ethics Statement

The animal study was approved by Washington University Animal Studies Committee (protocol number 2011134).

### Reagents and Instruments

All the chemicals were purchased from Sigma-Aldrich (St. Louis, MO). Isoelectric focusing (IEF) unit was the Bio-Rad PROTEAN IEF Cell. Spectrophotometer Plate Reader capable of reading 384-well plates with a wide wavelength selection and fluorescence reading was the SPECTRAMax Plus from Molecular Devices (Sunnydale, CA). Semi-Blot unit for protein transfer was Bio-Rad’s Trans-Blot SD Semi-Dry Transfer Cell. Frozen beef liver tissue was purchased from the local supermarket.

### Beef Liver Protein Preparation

Frozen beef liver tissue (5 grams) was chopped up with a razor and homogenized in 2.5 volumes of 50 mM Sodium Phosphate with 0.005% Beta-mercaptoethanol using a disposable plastic homogenizer. After spinning at 14,000 g for 15 minutes, the supernatant was collected and frozen at -20°C in small aliquots for single use.

### Protein Recovery Efficiency by the PEP

Protein recovery from the PEP plate and the Master Plate were calibrated by *E*. *coli* Host Cell Protein ELISA kit (Array Bridge, St. Louis, MO). Different concentrations of *E*. *coli* soluble protein extract were added in triplicates to the PEP plate and Master Plate respectively. After a two hour incubation at room temperature which mimicked the actual PEP procedure, the protein solution was recovered from the PEP and Master Plate and the amount of remaining *E*. *coli* protein in the solution was determined by *E*. *coli* Host Cell Protein ELISA kit according to the manufacturer’s protocol. The recovery rate was equal to dividing the protein concentrations remained by the starting protein concentrations.

### Drug Treatment and Mice Cochleae Protein Extraction

Two-month old CBA mice (20 for each gender), were used for this study. In the drug group, ten males and ten females were treated with 200 mg/kg zonisamide right before 2 hours, 96 dB (8–16 kHz octave band) noise exposures. The rest of the mice treated were with 0.9% NaCl as the control group. Cochleae from both groups were harvested 24 hours after noise exposure and collected in cold PBS on ice. The tissue were then snapped frozen on dry ice and stored at -80°C before protein extraction. For protein extraction, the cochleae were suspended in 1 ml of extraction solution containing 8 M urea and 2% CHAPS and transferred to a 35 ml sterile conical tissue grinder (VWR catalog number: 47732–448). After grinding for 5 min. on ice, the cochleae were transferred to a centrifuge tube and centrifuged for 10 min at 16,000 x g. The protein concentrations in the supernatant were determined from both the control group and drug-treated group.

For running the 2-D gel, IPG strips (18 cm, Bio-Rad) were rehydrated with 352 μl rehydration solution (350 μl of protein extract plus 2 μl of Bio-Lyte; pH 3–10 Bio-Lyte from Bio-Rad) at room temperature overnight.

### Assay Procedure

The assay was performed with the Array Bridge (www.arraybridge.com, St. Louis, Missouri) NADH Oxidase Landscape and NADPH Oxidase Landscape Kits (catalog number: AB000502 and AB000503 respectively). Before the assay, diluted reagents and buffers were allowed to reach room temperature (18°C–25°C). The link to the demonstration of the PEP technology is: http://www.youtube.com/watch?v=Hi45_ZcN5BY, and the link to the PEP experimental demonstration is: http://www.youtube.com/watch?v=DkiSXBkxO6s. The following is the essential steps in carrying out the PEP-based functional analysis:

1. Rehydrate the two protein samples (control and drug-treated) in IEF rehydration solution in 18 cm IPG strips overnight.
2. Run IEF using the recommended program from Bio-Rad (0–10,000 V in 4 Hrs. stay at 10,000 overnight until the refolding step with VHrs reaching at least 40,000.
3. Take out the two IPG strips and refold in a two-step process: step one, refolding in a specific refolding solution developed by Array Bridge that contains the cysteine-cysteine system, 14 most common elements found in the cell and all the common amino acids. The rationale of using the 14 elements is that many enzymes use metal elements as cofactors, during the IEF under refolding conditions, the metal elements were separated from the enzyme. This could be one of the reason enzymes lose activity after gel electrophoresis. During the first refolding step, the elements were added to assist the enzyme refolding process. It was also found that the addition of amino acids improved the refolding process as some of the amino acids were known to stabilize proteins and had been used in protein drug formulation. This step also allows for the diffusion of urea from the IPG strips. In step two, the IPG strips were transferred to a gel electrophoresis transfer buffer (Tris-Glycine with 0.1% SDS). In this step, urea continues to diffuse out from the IPG strips and more importantly, SDS will bind to the refolded enzyme and improve the resolution in the second dimension gel electrophoresis.
4. After the refolding process, the IPG strip was loaded onto a second-dimension gel and run directly without adding agarose solution as in typical 2-D gels. This modification is made to minimize the heat-induced enzyme inactivation by the hot agarose solution. The second dimension gel was run first at 80 V for 10 min followed by 120 V and up until the dye front was 1 cm from the bottom of the gel.
5. Carefully take out the gel and lay on top of the large format PEP plate which has been filled with PEP elution solution. The elution solution contains a large molecule enzyme stabilizer to minimize enzyme inactivation and prevent diffusion of eluted enzymes in each well. The elution was carried out in a semi-dry Trans-Blot device (Bio-Rad) at 20 V for 60 min.
6. After protein elution, the solution from each of the PEP plates were transferred to Master Plate using multiple transfer pipettes. The Master Plate is filled with 50 μl/well of selected buffer that is suitable for a specific enzyme assay. In this study, PBS was used in the Master Plate.
7. Transfer 25 μl enzyme solution from each well in the Master Plate and add to the enzyme assay plate. Add enzyme substrate and read 4 data points: 0, 60 min., 120 min. and overnight. The time-dependent increase of enzyme activity will help to eliminate false readings for the assay.
8. Calculate the enzyme activity by subtracting the OD 340 nm reading at different time point from the 0 min reading and use Microsoft Excel to construct the 3-D enzyme activity map.
9. For those wells with significant enzyme activity, the samples from the Master Plate were recovered and identified through mass spectrometry.

### Mass Spectrometry for Protein Identification

The entire amount of each submitted sample was separated on a 10% Bis-Tris Novex mini-gel (Invitrogen) using the MES buffer system. The gel was stained with coomassie and the entire mobility region was excised. Gel pieces were processed using a robot (ProGest, DigiLab) with the following protocol:

1. Washed with 25mM ammonium bicarbonate followed by acetonitrile.
2. Reduced with 10mM Dithiothreitol at 60°C followed by alkylation with 50mM iodoacetamide at RT.
3. Digested with trypsin (Promega) at 37°C for 4h.
4. Quenched with formic acid and the supernatant was analyzed directly without further processing.

The digests were analyzed by nano LC/MS/MS with a Waters NanoAcquity HPLC system interfaced to a ThermoFisher Q Exactive. Peptides were loaded on a trapping column and eluted over a 75μm analytical column at 350nL/min; both columns were packed with Proteo Jupiter resin (Phenomenex). A 30min gradient was employed. The mass spectrometer was operated in a data-dependent mode, with MS and MS/MS performed in the Orbitrap at 70,000 FWHM and 17,500 FWHM resolutions, respectively. The fifteen most abundant ions were selected for MS/MS.

Data were searched using a local copy of Mascot with the following parameters:

Enzyme: Trypsin
Database: SwissProt Human (concatenated forward and reverse plus common contaminants)
Fixed modification: Carbamidomethyl (C)
Variable modifications: Oxidation (M), Acetyl (Protein N-term), Deamidation (NQ), Pyro-Glu (N-term Q)
Mass values: Monoisotopic
Peptide Mass Tolerance: 10 ppm
Fragment Mass Tolerance: 0.015 Da
Max Missed Cleavages: 2
Mascot DAT files were parsed into the Scaffold software for validation, filtering and creating a nonredundant list per sample. Data were filtered using a minimum protein value of 90%, a minimum peptide value of 50% (Prophet Scores) and requiring at least two unique peptides per protein.

## Results

### Optimizing Transfer Conditions of the PEP Technology

The first step in developing our PEP technology was to define optimal conditions for the effective transfer of active proteins from the 2-DE gel into the PEP. In a typical 2-DE, reducing reagents such as β-mercaptoethanol or dithiothreitol (DTT) are used to reduce disulfide bonds in order to improve the separation efficiency in the presence of a high concentration of urea (normally 8 M). Because our purpose was to separate proteins efficiently while retaining their biological activities, two main modifications were made for the 2-DE process. First, no reducing reagent was used in the samples during IEF to keep the disulfide bonds intact in proteins. Second, a reduced SDS concentration was used for the IPG strip incubation before running the second dimensional gel. Instead of the typical 2% SDS, the SDS concentration was reduced 20-folds to 0.1%. Preliminary experiments showed that, at 0.1% SDS concentration, sample proteins can still be separated efficiently, and most importantly, the testing enzyme (Horse Radish Peroxidase) was still active (data not shown), consistent with previous publications showing that a large number of enzymes including protein kinases, phosphatases, proteases and many other enzymes are active in the presence of a detergent such as SDS [17].

After the protein separation in the 2-DE, sample proteins were transferred to the PEP by electroelution. To determine the protein transfer efficiency, two separate gels with and without electroelution were stained for protein detection. After electroelution, most of the proteins had been transferred as only very weakly stained bands were detected in the gel after the electroelution while strong stained bands were detected in the gel without the electroelution (data not shown).

### Protein Recovery from the PEP Plate and Master Plate

After establishing the optimal transfer conditions, the protein recovery efficiency from the PEP plate was determined. Because low-level proteins can be lost by binding to the plastic surface and the fact that the molecular sieve membrane and the plate grid of the PEP could potentially bind the proteins eluted from the 2-DE gel, it was important to determine the efficiency of protein recovery at different concentrations. Because some of the 2-DE gel protein spots only exist at low nanogram (ng) levels whereas others could exist at microgram (μg) levels, three different protein concentrations (4 ng/well, 400 ng/well and 4000 ng/well) which reflect the range of typical 2-DE gel protein spot abundance were used to test protein recovery from the PEP. Furthermore, to test proteins with different sizes and different isoelectric points, a protein mixture prepared from *E*. *coli* cellular proteins were tested and a quantitative ELISA method was used to measure proteins recovered from the PEP. Preliminary tests showed that protein mixtures ranging from 4 ng/well to 4000 ng/well were recovered efficiently from the PEP, which led to the following functional assays.

**Fig 1:**
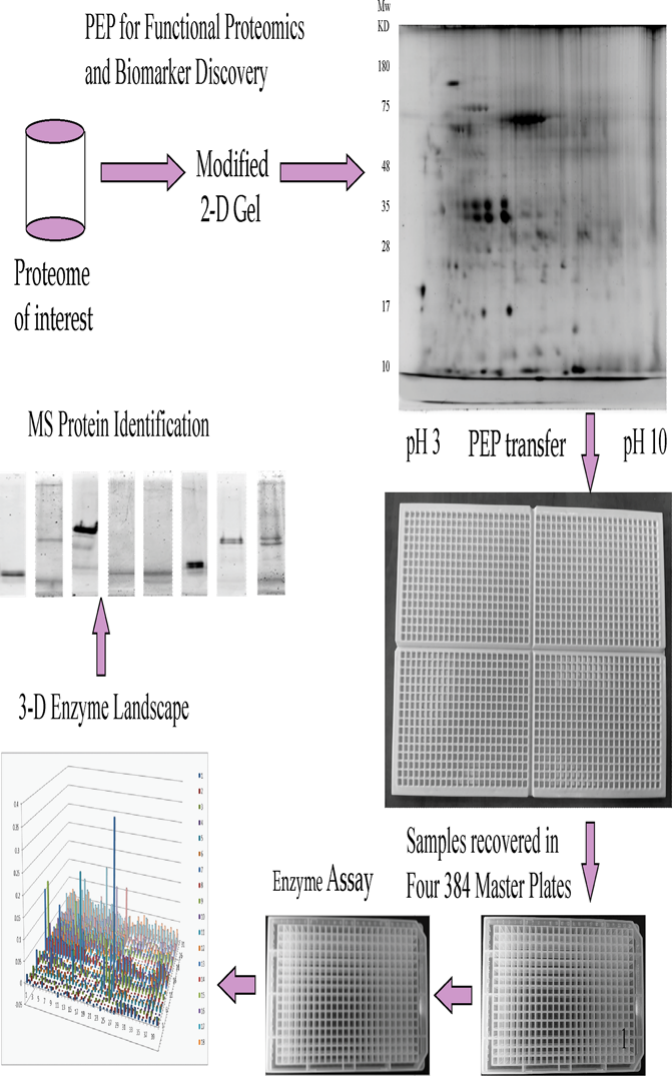
Diagram of the PEP Technology with MS protein identification.

After functional protein assays were carried out in separate 384-well microplates (Fig. 1), the next step in the development of the PEP was to demonstrate if proteins transferred to the Master Plate could be successfully transferred to enzyme assay plates for functional assays. In the current setting, protein mixtures at levels of 10 ng/well, 100 ng/well and 1,000 ng/well were tested for recovery from the master plate after an incubation of 4 hours. It was found that a significant amount of proteins could be recovered from the Master Plate, especially at levels typically seen in the 2-DE gel (100 ng/well-1,000 ng/well). Given the current detection sensitivity of mass spec technology, this should be suitable for protein characterization and identification.

### NADH and NADPH-Dependent Oxidase Activity Analysis from Beef Liver

**Fig 2:**
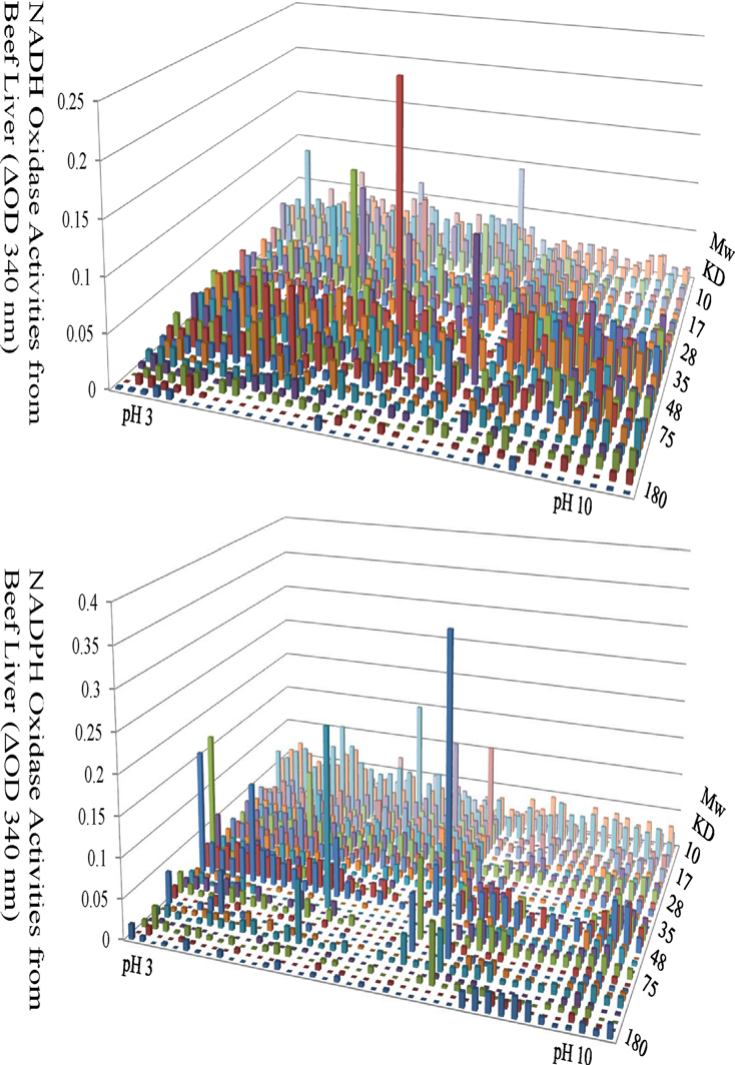
Enzymatic Assays of NADH- and NADPH-dependent Oxidases from the Beef Liver Proteome. The functional landscape of beef NADH (top panel) and NADPH (bottom panel) dependent oxidases from were shown as a proof of principle of the PEP technology. These oxidases were separated by 2-D electrophoresis based on their unique isoelectric points (X axis) and molecular weight (Y axis). Their activities were shown as the ability to oxidize NADH or NADPH, respectively (Z axis).

Before using this new PEP method to analyze oxidation pathways in the mouse cochlea, we did additional testing with protein samples from beef liver since beef liver is very rich with metabolic enzymes. Cellular proteins prepared from beef liver by the PEP technology were used to optimize conditions for the systematic analysis of NADH and NADPH oxidases. As shown in Fig. 2, a large number of NADH and NADPH-dependent oxidase activities were detected across the 2-DE landscape with different molecular sizes and isoelectric points. A large dimension gel was used to increase the loading capacity and assay sensitivity. 2 mg of total liver proteins were loaded for 2-DE gel separation and subsequent functional analysis. A special substrate mixture was designed to cover as many redox enzymes as possible. The substrate mixture was composed of all the 20 amino acids and several important metabolic sugars that are most commonly used in cell metabolism.

The functional landscape of beef NADH (top panel) and NADPH (bottom panel) dependent oxidases from were shown as a proof of principle of the PEP technology. These oxidases were separated by 2-D electrophoresis based on their unique isoelectric points (X axis) and molecular weight (Y axis). Their activities were shown as the ability to oxidize NADH or NADPH, respectively (Z axis).

### Response of Redox Enzymes to Drug Treatment

**Fig 3:**
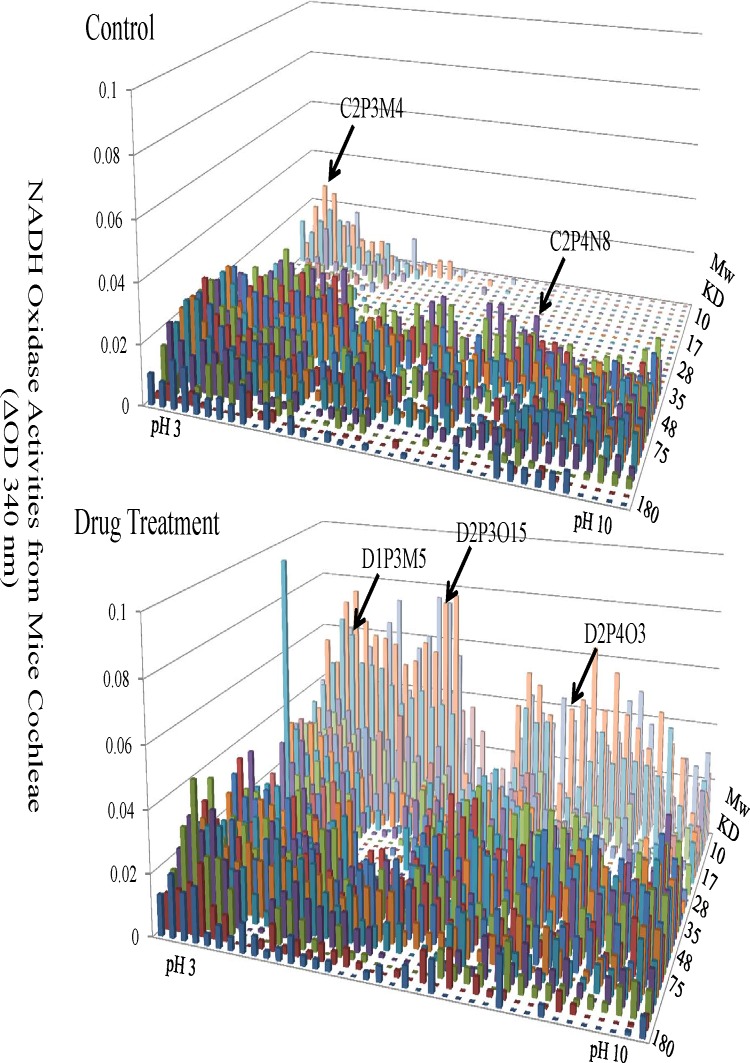
Changes of NADH-dependent Oxidase Activities in Mouse Cochleae from the Control and Drug Treatment Groups. The functional landscape of NADH-dependent oxidases from mouse cochleae with (bottom panel) or without drug treatment (top panel) were shown. These oxidases were separated by 2-D electrophoresis based on their unique isoelectric points (X axis) and molecular weight (Y axis). Their activities were shown as the ability to oxidize NADH (Z axis).

After the optimization of our PEP technology, this method was applied to the study molecular mechanisms underlying prophylactic functions of zonisamide against noise-induced hearing loss in mice. Total proteins (2 mg) extracted from cochlear tissues were separated by a large dimensional 2-DE gel (16 cm wide, 15 cm height), and large format PEP was used to carry out protein elution and analysis. Fig. 3 showed the NADH-dependent oxidase activities from the control and drug treated groups respectively. There was a dramatic shift of redox enzyme activities after the drug treatment, especially for enzymes with low molecular weights.

The functional landscape of NADH-dependent oxidases from mouse cochleae with (bottom panel) or without drug treatment (top panel) were shown. These oxidases were separated by 2-D electrophoresis based on their unique isoelectric points (X axis) and molecular weight (Y axis). Their activities were shown as the ability to oxidize NADH (Z axis).

### Protein Purification and Mass Spectrometry for Protein Identification

**Fig 4:**
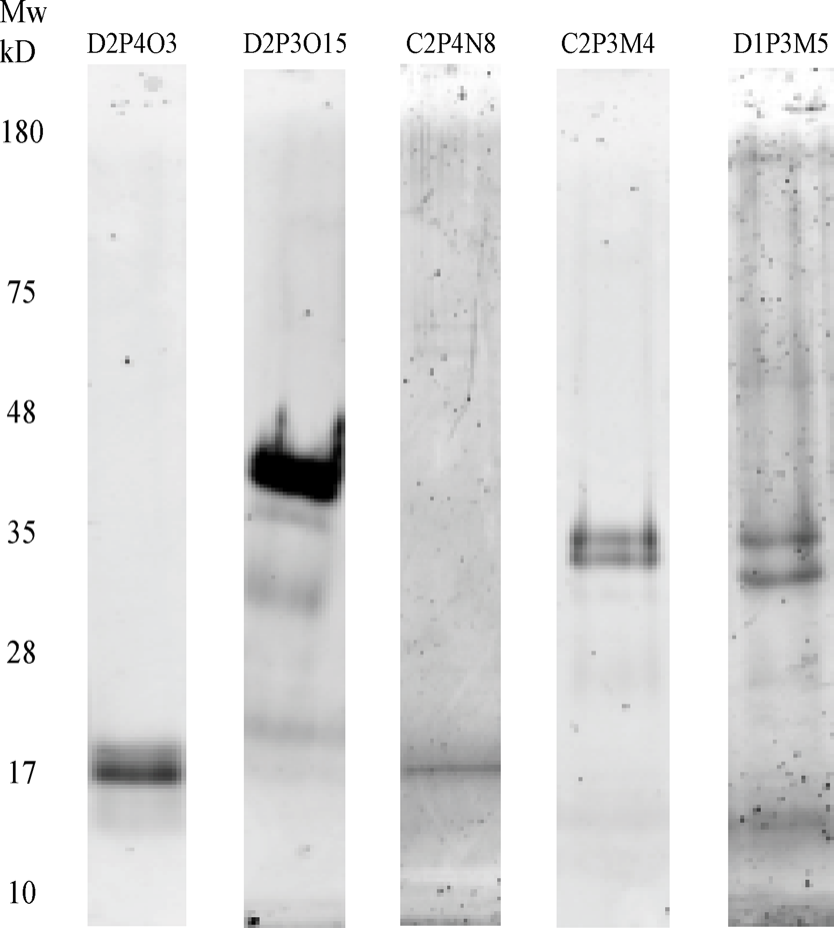
SDS-PAGE Analysis of Proteins Recovered from the Individual PEP Wells. Five samples with significant oxidase activity were selected, two from the control plate and three from the drug treated plate, and analyzed by SDS-PAGE. Only one or two major protein bands were detectable for each sample.

A portion of the samples from wells with relatively high redox enzyme activities were selected for protein purity and mass spectrometry analysis. As demonstrated in Fig. 4, many of the wells with redox enzyme activity had just one or two major protein species. It should also be noted that because no reducing reagent was used for the sample treatment after isoelectric focusing, enzymes with heterogeneous subunits or enzyme complexes with interchain disulfide bonds were not disrupted in the current separation process, partly explaining why some of the wells tested have more than one protein species.

Five samples with significant oxidase activity were selected, two from the control plate and three from the drug treated plate, and analyzed by SDS-PAGE. Only one or two major protein bands were detectable for each sample.

After the redox enzyme activity analysis, a selected number of fractions with relatively higher enzyme activities were submitted for mass spectrometry protein identification. As listed in Table 1, five redox enzymes were identified from the samples submitted.

**Table 1:** NADH oxidases identified by mass spectrometry after separation with PEP technology.

Sample Number	Sample Location On PEP*	Tentative Protein ID
Sample-1	D2P4O3	Glyceralde-3-phosphate Dehydrogenase
Sample-2	D2P3O15	Glyceralde-3-phosphate Dehydrogenase
Sample-3	C2P4N8	Malate Dehydrogenase
Sample-4	C2P3M4	L-lactate Dehydrogenase
Sample-5	D1P3M5	Glyceralde-3-phosphate Dehydrogenase

* The location of each protein on the 2-D gel was defined by three parameters: the treatment group, the plate number and the location on the 384-plate. 1. The treatment group is represented in the two letters, D2 represents drug treatment experiment-2, and C2 represents control group-2. 2. The large format PEP is composed of 4 384-well microplates, upper left is plate-1 (P1), upper right is P2, lower left is P3 and lower right is P4. For the sample location letter 3 and 4, P4 represents Plate-4. The last two letters represent the sample location on the plate, O3 represents row “O” column 3.

## Discussion

New tools developed for studying system biology will facilitate our understanding of complex biological processes. Because of the advancements in systems biology, global gene expression profiles can be analyzed by DNA chip or next generation sequencing. At the protein level, the proteome can be studied based on relative protein abundance and post-translational modifications. RNAi and the recently developed CRISPR technology can be used to down-regulate or knock-out genes from a genome systematically [18–20]. All these advancements provide valuable insight on cell metabolism and other important biochemical processes. The recent publications of the human proteome by two separate groups present a major advancement in our understanding of the human proteome and shed new light on protein regulation in human cells [21,22]. After the completion of the human proteome, the next question is to develop effective ways for functional studies of the proteome. Because of extensive post-translational protein modification, proteins with the same abundance could have very different activity status. For example, many protein kinases can be inactivated by dephosphorylation. Therefore, it is important to not only consider gene expression and protein abundance, but also the protein functional landscape.

This study focused on the development of a new technology that can systematically analyze protein functions of a whole enzyme family or the entire proteome. The functional information obtained from this technology should be complementary to those acquired through the analysis of gene expression and protein abundance. From a drug discovery point of view, the functional map of a proteome more accurately reflects the actions inside the cell because it is the activities of enzymes and functional proteins that drive cellular processes.

During drug development, two major challenges are frequently encountered. One is to select the right drug target(s) and the other is to develop a molecule with high specificity toward the target without interacting with other important protein functions. The PEP technology can prove to be valuable to address these two challenges. First of all, it is known that many diseases such as hearing loss are driven by multiple genes and pathways. Getting a clear picture of how protein functions have been changed in drug-treated cells as compared to untreated cells will provide insights on whether right proteins or pathways are targeted. Secondly, if a drug candidate is identified, it will be important to find out whether this molecule can inhibit other enzymes in the same family i.e. off-target interactions. For example, protein kinases are important targets for many diseases. However, serious side effects were observed in many of the protein kinase inhibitors that were developed. Could this be the results of off-target interactions with other protein kinases? A PEP-based protein kinase analysis could show how many protein kinases are inhibited by this kinase inhibitor other than the intended target. Another area of potential application for the PEP technology is to target metabolic enzymes that are indicative of cancer. As of late, there has been a resurgence in finding targets and developing appropriate molecules focusing in cancer metabolism [23–27]. However, there are hundreds of enzymes involved in cell metabolism and many share the same cofactor such as NADH or NADPH. Is it possible to select the right drug candidate using the PEP technology and analyze an entire family of metabolic enzymes from a proteome as demonstrated in this current report? Furthermore, fig. 2 indicated that the PEP technology can detect activity changes of numerous redox enzymes from a proteome. Combined with the fact that redox activity can reflect the overall metabolic status of the cell, this readout could provide a systematic landscape of the cell redox status and facilitate the identification of key enzymes through mass spectrometry.

In the current study, the effect of a specific drug on mice cochlear redox enzymes was analyzed. As shown in Fig. 3, there are dramatic changes in the pattern of redox enzymes in enzyme distribution and activity level upon drug treatment. In this case, instead of studying one or a few enzymes from the drug treatment, the whole proteome was interrogated with the drug treatment. In addition, other enzyme families such as protein kinases, phosphatases, and proteases can also be analyzed in the same way. Because of gene editing and post-translational modifications, almost all functional proteins, including enzymes, have isoforms. The PEP technology is a valuable tool in the analysis of the function of any enzyme isoforms to further understand their biochemical functions. As demonstrated in Table 1, the same enzyme was identified from different positions on the 2-DE gel, suggesting that the enzyme (GDH) exists as isoforms or is associated with different proteins in the cell. An extensive identification of all the enzymes with redox activity will provide a comprehensive landscape that can be used in further understanding how zonisamide prevents noise-induced hearing loss.
